# Microbiome-macrophage crosstalk in the tumor microenvironment: implications for oral squamous cell carcinoma progression and therapy

**DOI:** 10.3389/fimmu.2025.1651837

**Published:** 2025-08-29

**Authors:** Xin Deng, Shaohong Huang

**Affiliations:** Stomatological Hospital, School of Stomatology, Southern Medical University, Guangzhou, China

**Keywords:** oral squamous cell carcinoma, tumor-associated macrophages, microbiota, inflammation, chemokine receptors, immune suppression

## Abstract

Oral squamous cell carcinoma (OSCC) remains a formidable malignancy with persistently poor clinical outcomes. Recent research has underscored the pivotal role of the innate immune system, particularly tumor-associated macrophages (TAMs), a key component of the myeloid lineage, in orchestrating the tumor microenvironment (TME) and shaping disease progression. As professional phagocytes of the innate immune system, macrophages not only mediate pathogen recognition and inflammatory responses but also undergo functional polarization in response to local cues. In OSCC, dysbiosis of the oral microbiota, marked by the overrepresentation of species such as *Fusobacterium nucleatum* and *Porphyromonas gingivalis*—acts as a chronic inflammatory trigger that promotes epithelial-mesenchymal transition (EMT), immune evasion, and tumor growth. These pathogenic bacteria actively engage innate immune signaling pathways such as TLRs and CSF-1R, skewing macrophages toward an immunosuppressive M2 phenotype. M2-like TAMs then contribute to tumor progression by secreting anti-inflammatory cytokines (IL-10, TGF-β), promoting angiogenesis, and expressing immune checkpoint ligands such as PD-L1. This review summarizes current knowledge on the bidirectional crosstalk between dysbiotic microbiota and innate immune macrophages in OSCC, highlighting key receptor-mediated pathways and their implications for immune suppression, metastasis, and therapy resistance. Targeting microbiota modulation or innate immune reprogramming represents a promising strategy for restoring anti-tumor immunity and enhancing therapeutic efficacy in OSCC.

## 1 Introduction

Oral squamous cell carcinoma (OSCC) accounts for 90% of oral malignancies and remains the predominant head and neck cancer subtype (1–3). Despite surgical resection being the standard of care, therapeutic options are limited, and the global 5-year survival rate remains below 50% (4). Thus, further research into the etiopathogenesis and mechanisms driving OSCC is essential. Since the WHO classification of *Helicobacter pylori* as a carcinogen, interest in bacteria–cancer associations has intensified, revealing diverse molecular mechanisms (5). The oral cavity harbors over 700 bacterial species, making it one of the most complex microbial ecosystems in the human body (6, 7). Although the link between oral microbiota and OSCC is debated, prevailing hypotheses posit that pathogenic bacteria directly promote oncogenesis or that oral dysbiosis accelerates tumor progression (8).

Beyond microbial influences, immune regulation is pivotal. Macrophages—key effectors of innate immunity—mediate pathogen clearance, modulate inflammation, and orchestrate tissue repair (9). Tumor-associated macrophages (TAMs), a dominant immune population within the OSCC stroma, critically shape the tumor microenvironment (TME) (10). TAMs display functional plasticity: the pro-inflammatory, anti-tumor M1 phenotype contrasts with the TME-induced M2 phenotype, which fosters invasion, metastasis, and immunosuppression (11, 12). In OSCC, TAMs express receptors such as CD206, CD163, and Toll-like receptors, as well as cytokine and chemokine receptors including IL-1R, CSF-1R, and CCR family members, which serve as biomarkers for TAM quantification and tumor dissemination (13, 14). This review summarizes current evidence on OSCC-associated oral microbiota shifts, TAM–tumor cell interactions mediated by surface receptors, and the emerging role of microbiota in modulating macrophage function to drive OSCC pathogenesis.

## 2 Microorganisms in OSCC

In OSCC, microbial populations vary significantly across tumor stages and oral sites. YANG et al. (8) analyzed microbial composition in adjacent normal tissues, tumor tissues, and saliva of OSCC patients, finding *Streptococcus* and *Peptostreptococcus* species most abundant in tumor tissues, while *Neisseria* species were prevalent in saliva. Similarly, SARKAR et al. (15) observed that *Prevotella* and *Fusobacterium* species were more abundant in tumor tissues compared to normal tissues. Li et al. (16) reported significant microbial differences between healthy individuals and OSCC patients, with *Porphyromonas* and *Peptostreptococcus* species enriched in tumor samples. Notably, *Porphyromonas* abundance correlated with elevated C-reactive protein levels in OSCC patients. Additionally, oral microbiota correlates with clinical tumor staging: *Treponema* species are abundant in early-stage tumors, while *Moraxella* species dominate advanced-stage tumor tissues (8). Periodontal disease is a recognized OSCC risk factor (17), linking oral microbiota-induced inflammation to cancer development. ZHANG et al. (18) confirmed increased *Fusobacterium nucleatum* abundance in OSCC, with *F. nucleatum* showing progressive enrichment from normal tissues to cancerous tissues (8, 19). *Porphyromonas gingivalis* is also associated with OSCC (20, 21), and higher abundance of *Fusobacterium*, *Peptostreptococcus*, and *Prevotella* species is found in tumor tissues compared to gingival squamous cell carcinoma and periodontal disease (22). Beyond these well-characterized taxa, other microorganisms, including Treponema denticola and Campylobacter rectus, have also been detected at increased abundance in the oral cavity of OSCC patients and are thought to contribute to chronic inflammation and immune modulation, potentially influencing tumor initiation and progression.

In oral cancer, microorganisms induce inflammation by producing cytokines and chemokines, promoting tumor cell proliferation and epithelial-mesenchymal transition (EMT), which enhances migration and invasion abilities (20, 23). Chronic infections with *F. nucleatum* and *P. gingivalis* increase the severity of tongue tumors, with experimental tumors in mice being approximately 2.5 times larger than controls (23, 24). *F. nucleatum* promotes OSCC progression and is associated with poor prognosis, enhancing tumor cell proliferation, migration, and immune regulation in the TME (23, 25, 26). It regulates EMT through the lncRNA MIR4435-2HG/miR-296-5p/Akt2/SNAI1 signaling pathway (27), and activates STAT3 in CRC to upregulate EMT genes (28). *P. gingivalis* activates ERK1/2-Ets1, p38/HSP27, and PAR2/NF-κB pathways, promoting matrix metalloproteinase (MMP) expression and tumor invasiveness (29). Both *F. nucleatum* and *P. gingivalis* activate the TLR/MyD88-triggered integrin/FAK pathway, enhancing tumor invasiveness (30). Microorganisms play a critical role in exacerbating oral diseases and potentially in the development and progression of oral cancer (31).

## 3 Macrophages in OSCC

### 3.1 Role of macrophages in tumors

TME comprises diverse non-malignant components, including fibroblasts and immune cells, that collectively shape tumor biology (32, 33). TAMs, through the secretion of cytokines, chemokines, and growth factors, exert profound influence on tumor initiation, progression, and metastasis (34). Exhibiting notable phenotypic plasticity, macrophages polarize along a continuum from classically activated M1 to alternatively activated M2 states (35–37). M2 macrophages are subclassified into M2a (IL-4/IL-13–induced), M2b (immune complex/LPS–induced), M2c (glucocorticoid/IL-10–induced), and M2d (IL-6/adenosine–induced), each performing specialized functions in OSCC (38, 39). M2a macrophages promote wound healing and extracellular matrix (ECM) deposition, facilitating tissue remodeling (40, 41). M2b cells generate both pro- and anti-inflammatory cytokines, establishing an immunoregulatory milieu (42, 43). M2c macrophages mediate potent immunosuppression via IL-10 and TGF-β secretion, supporting tumor survival (44). Importantly, M2d macrophages—also referred to as tumor-associated macrophages with an “angiogenic” phenotype—are particularly relevant to OSCC because they release high levels of vascular endothelial growth factor (VEGF), platelet-derived growth factor (PDGF), and matrix metalloproteinases (MMP−9 and MMP−2) (45–47). By contrast, M1 macrophages, though less well classified and capable of subtype interconversion, dominate early tumor stages, releasing TNF-α, CXCL9, CXCL10, iNOS, and ROS to induce inflammation and eliminate tumor cells (48–51). M2 phenotypes predominate in advanced disease, sustaining chronic inflammation, ECM remodeling, migration, invasion, and neovascularization via PDGF, TGF-β, and related mediators (34, 52). Among these, M2a, M2c, and M2d subtypes are particularly enriched in TAM populations, functioning as principal drivers of OSCC progression (53, 54).

### 3.2 TAM surface receptors in OSCC progression

#### 3.2.1 Human mannose receptor CD206 and CD163

Human mannose receptor CD206 is an endocytic transmembrane protein containing multiple carbohydrate-binding domains. Its ligands can include bacterial products, tumor metabolites, or other synthetic proteins (55). Structural modulation of CD206 using the synthetic ligand RP-182 suppresses tumor growth, prolongs survival, and reprograms M2 macrophages toward an M1 phenotype through endocytosis, phagolysosome formation, and autophagy (56). In OSCC, plasminogen activator inhibitor-1 (PAI-1) and IL-8 inhibit monocyte differentiation into CD206^+^ TAMs (57). CD206^+^ TAMs secrete higher epidermal growth factor (EGF) levels than CD163^+^ or CD204^+^ TAMs, enhancing OSCC proliferation and invasion via EGF receptor signaling, an effect abrogated by EGFR blockade (58). Through STAT3 activation, CD206^+^ M2 TAMs sustain a pro-tumor milieu, releasing VEGF, TGF-β, EGF, urokinase-type plasminogen activator (uPA), and multiple matrix metalloproteinases (MMPs), thereby promoting tumor growth, immune suppression, angiogenesis, metastasis, and chemoresistance (59). CD163, widely applied as an immunohistochemical TAM marker, is associated with tumor infiltration and invasion, with expression levels correlating with tumor stage, nodal involvement, and metastasis (60). Functionally, CD163^+^ TAMs foster tumor immune evasion; their depletion in melanoma models increases cytotoxic T cell infiltration and inflammatory monocyte recruitment, markedly enhancing tumor regression (61). In OSCC, co-expression of CD163 and CD204 in M2 TAMs coincides with IL-10 secretion and PD-L1 expression, potentially suppressing T cell function and facilitating invasion and metastasis (62). Moreover, PFKFB3 expression positively correlates with CD163 levels in OSCC, implicating this glycolytic regulator in TAM-mediated angiogenesis (63). Targeting CD163 may thus attenuate tumor-induced immunosuppression.

#### 3.2.2 The role of colony-stimulating Factor 1 receptor

Colony-stimulating factor receptors (CSFR) are essential for the differentiation of myeloid stem cells into monocytes and macrophages. Chemoresistant colorectal cancer cells release CSF, which binds to CSFR, recruiting TAMs and upregulating PD-L1, leading to chemotherapy resistance and poor prognosis (64, 65). Pharmacological inhibition of CSF1R has been shown to reprogram TAMs from an M2-like immunosuppressive phenotype toward an M1-like pro-inflammatory state, thereby restoring antitumor immunity (66). A large-scale OSCC study found that CSF-1 levels correlated with TAM infiltration, and CSF-1 signaling blockade with BL2945 not only inhibited OSCC growth but also significantly reduced CSF-1 expression and TAM infiltration (67). These findings suggest that CSF-1, abundantly produced by OSCC cells and surrounding stromal components, binds chemotactically to CSF1R on TAMs, thereby fueling tumor cell proliferation. Moreover, in oral cancer, CSF-1 co-expresses with the transcriptional activator TWIST1 of epithelial-mesenchymal transition (EMT), accompanied by TAM infiltration, suggesting that CSF-1 induces TAM chemotaxis through CSFR activation, enhancing OSCC’s epithelial-mesenchymal transition and invasion (68). Therefore, targeting the CSF-1 receptor on TAMs represents an important strategy for combating tumor growth (**Table 1**).

**Table 1:** Summary of major TAM surface receptors in OSCC.

Receptor	Major Ligands	Key Signaling Pathways	Immune Function	Target
CD206 (mannose receptor)	Mannose-containing glycans, microbial antigens	Endocytosis, STAT3 activation	Promotes M2 polarization, angiogenesis, immune suppression	Conformation-modifying molecules (e.g., RP-182) to repolarize TAMs
CD163 (scavenger receptor)	Hemoglobin-haptoglobin complexes	JAK/STAT and anti-inflammatory pathways	Enhances immune suppression, tumor invasion, and angiogenesis	Targeting CD163+ TAMs to relieve T-cell suppression
CSF-1R	CSF-1, IL-34	CSF-1/TWIST1-driven EMT	TAM recruitment, M2 polarization, PD-L1 upregulation, EMT	CSF-1R inhibitors to block TAM recruitment and reprogram TAMs
Toll-like receptors (TLRs)	Pathogen-associated molecular patterns (LPS, HMGB1)	MyD88/NF-κB, TRIF pathways	Context-dependent: TLR4 promotes tumor growth; TLR7/8 agonists induce M1 polarization	TLR agonists or modulators to repolarize TAMs
IL-1R	IL-1α, IL-1β	MyD88/NF-κB, MAPK	Induces M2 polarization, enhances chemokine secretion (CXCR4/SDF-1α)	IL-1R antagonists to limit M2 skewing and tumor invasion
Chemokine receptors (CXCR4)	CXCL12/SDF-1	CXCL12/CXCR4 axis	Monocyte recruitment, M2 polarization, tumor stemness	CXCR4 inhibitors to prevent TAM infiltration and reduce immune suppression

#### 3.2.3 The role of toll-like receptors

Toll-like receptors (TLRs), including TLR2, TLR4, TLR7, TLR8, and TLR9, are critical pattern recognition receptors on TAMs, with diverse ligands such as tumor proteins, acute-phase proteins, drugs, and bacterial metabolites. TLR4 signaling promotes tumor growth, while TLR7, TLR8, and TLR9 signaling may exert anti-tumor effects (69). Ferroptosis, a regulated cell death driven by lipid peroxidation, serves as a tumor-suppressive mechanism (70). Tumor cells undergoing ferroptosis release HMGB1, which binds to TLR4 on myeloid cells, enhancing differentiation into mature cells (71). Activation of TLR4/NF-κB signaling through carbon nanotubes induces M2-polarized TAMs to shift to M1, inhibiting metastasis in mouse models (72). Alpha 1-acid glycoprotein (AGP) activates TLR4 through CD14, modulating PD-L1 expression and IL-6 production, thus enhancing tumor immune suppression (73). Ginseng-derived nanoparticles (GDNPs) induce M2 to M1 polarization via TLR4/MyD88, promoting apoptosis in head and neck squamous cell carcinoma (74). In colorectal cancer, cathepsin K secretion disrupts the gut microbiome and activates TLR4 to promote M2 macrophages, accelerating progression (75). OSCC may follow similar mechanisms. Overexpression of ALDH3A1 in Cal27 cells inhibits IL-6 and suppresses TLR4 activation in TAMs, reducing inflammation (76). Oral administration of Dendrobium officinale polysaccharide (DOP) promotes M1 polarization via TLR2 on TAMs, inhibiting tumor growth (77). TLR7 and TLR8 agonists reverse TAMs from M2 to M1, reducing radiation resistance and tumor growth (78, 79). Immune-modulating agents delivered via polymer micelles targeting TLR7 reduce immune suppression in the TME (80).

#### 3.2.4 Interleukin-1 and chemokine receptors

The interleukin-1 receptor (IL-1R) is activated by its canonical ligands, IL-1α and IL-1β, whereas the interleukin-1 receptor antagonist competitively inhibits this signaling. Ligand binding to IL-1R predominantly triggers the MyD88–NF-κB and mitogen-activated protein kinase (MAPK) pathways. Accumulating evidence indicates that the IL-1R/MyD88 signaling axis regulates programmed death-1 (PD-1) expression, thereby sustaining the immunosuppressive activity of TAMs and facilitating tumor progression in melanoma and other malignancies (81, 82). OSCC cells release IL-1β to recruit monocytes, and IL-1R-mediated TAM conversion to the M2 phenotype imparts immune-suppressive capabilities (83). Following IL-1β-induced M2 polarization, TAMs continue to secrete IL-1β. OSCC and TAMs induce the expression of CXCR4 or stromal cell-derived factor-1 alpha (SDF-1α) through IL-1β/IL-1R signaling and hypoxia-induced activation of ERK signaling, thus promoting the expression of MMP-9 and MMP-13, which facilitate OSCC migration and invasion (84). Therefore, IL-1R modulation in TAMs could be a promising target for cancer therapy. Chemokine receptors are part of the seven-transmembrane G-protein-coupled receptor family, with many receptors present on TAMs. Tumor-associated fibroblasts (CAFs) in the microenvironment attract monocytes via CXCL12/CXCR4 signaling, which induces the polarization of TAMs to the M2 phenotype. These polarized TAMs contribute to the formation of tumor stem cells in OSCC, promoting proliferation, reducing apoptosis, and enhancing migration (85). This receptor family, with its large array of ligands, presents considerable research potential and could become a therapeutic target in TAM-based OSCC treatment.

#### 3.2.5 The role of other receptors

TAMs express numerous surface receptors, representing an area of active research for receptor-based therapies. Tumor-associated endothelial cells aberrantly express and secrete HSPA12B, which can be partially engulfed by macrophages through the oxidized low-density lipoprotein receptor 1 (OLR1), triggering PI3K/Akt/mTOR signaling and increasing the expression of M2 markers (86, 87). Immunohistochemical staining has revealed that TAMs in endogenous OSCC express more patched-1 (PTCH) receptors than in exogenous OSCC, with sonic hedgehog (SHH) ligands inducing tumor invasion through autocrine signaling and regulating OSCC stroma-parenchyma interactions via paracrine signaling (88). In addition, TAMs obtain CD73 from exosomes secreted by head and neck squamous carcinoma cells and express it on their cell membrane. This exosome-mediated transfer enhances immune evasion and tumor invasiveness through activation of CD73–NF-κB p65 signaling, and promotes the secretion of immunosuppressive mediators such as PD-1, PD-L1, and pro-inflammatory cytokines (89). Furthermore, microbial-derived tryptophan metabolites have been shown to activate the aryl hydrocarbon receptor on TAMs, thereby amplifying their immunosuppressive functions and further attenuating antitumor immunity (90).

## 4 Microbial and macrophage crosstalk in the development of OSCC

### 4.1 Crosstalk between oral microbiota and macrophages

The balance between oral microbiota, immune cells, and the epithelial barrier is crucial for maintaining oral microbiome stability. Disruption of this balance, caused by pathogenic microbes, can lead to inflammatory responses, mucosal barrier breakdown, and oral diseases, such as periodontitis, eventually promoting oral cancer. Periodontitis, the sixth most prevalent global disease (91), is influenced by bacterial infections and immune dysregulation. Key bacteria, including *P. gingivalis*, *F. nucleatum*, and *T. forsythia*, have been extensively studied in periodontitis models (92), with macrophages playing a significant role in disease progression. Macrophages polarize into M1 macrophages in response to periodontal pathogens, contributing to the inflammatory environment (93). *P. gingivalis* inhibits α-ketoglutarate (α-KG) expression, suppressing M2 macrophage generation and inducing M1 macrophage polarization to maintain inflammation (94). It drives M1 macrophage infiltration into deep periodontal tissues, promoting gingival inflammation and alveolar bone resorption (94, 95). Additionally, macrophages interacting with oral commensal bacteria, such as through IFN-γ/LPS stimulation, can enhance pathogen survival and disease progression (96). In the tumor microenvironment, oral microbiota promotes M2 polarization of macrophages. Periodontal pathogens activate IL-17^+^ γδ T cells and increase M2-type TAM infiltration in OSCC (97). *P. gingivalis* has also been shown to protect OSCC cells from macrophage phagocytosis and induce M2 macrophage polarization (98). Furthermore, T. forsythia produces outer membrane vesicles that activate NF-κB and upregulate TNF-α, IL-8, and IL-1β in macrophages (99), while *P. gingivalis* upregulates miR-155, promoting NLRP3 inflammasome activation and macrophage pyroptosis (100). Other less frequently discussed species, such as *Treponema denticola* and *Campylobacter rectus*, have also been reported to modulate immune responses in periodontal disease and may contribute to macrophage activation and polarization in the OSCC microenvironment, though their specific mechanisms remain less well characterized.

### 4.2 Impact of microbial and macrophage regulation on OSCC development

The imbalance between the oral mucosa, microbiota, and immune system leads to oral diseases. In germ-free mice, intestinal mucosal atrophy and reduced macrophage numbers recover after microbiota colonization, with full recovery by five weeks (101). In dysbiotic mice, macrophages release IL-6, promoting colorectal cancer proliferation and EMT, with macrophage depletion reversing tumor-promoting effects (102). Co-culturing *Faecalibacterium prausnitzii* with macrophages induces morphological changes in intestinal epithelial cells, highlighting microbiota-immune-epithelial regulation (103). Dysbiosis weakens the mucosal barrier, facilitating bacterial translocation and chronic inflammation, which leads to DNA damage, cytokine/chemokine production, and tumor proliferation, migration, and apoptosis inhibition (104). *Fusobacterium nucleatum* (*F. nucleatum*) plays a crucial role in OSCC, promoting M2-type TAM aggregation and immune suppression by inhibiting macrophages and T cells (105). *F. nucleatum* disrupts epithelial tight junctions and induces immune suppression via indoleamine 2,3-dioxygenase (IDO) upregulation (106). It activates the NF-κB/miR-132 axis, promoting M2 macrophage polarization and CRC metastasis, as well as TLR4/IL-6/p-STAT3/c-MYC pathways to enhance immunosuppressive effects (107–110).

**Figure 1:**
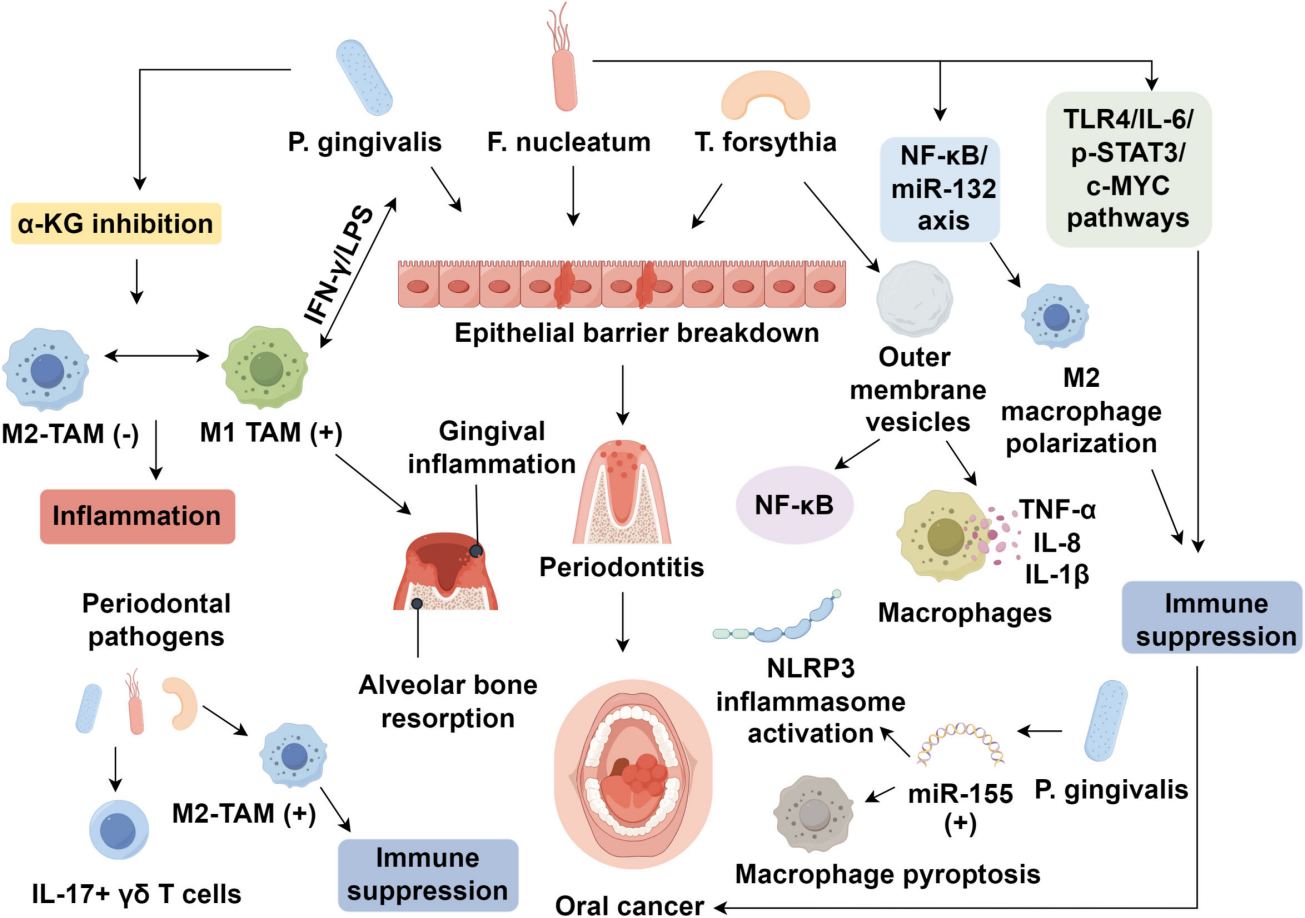
Microbiome-macrophage crosstalk in oral squamous cell carcinoma.

*P. gingivalis* also induces M2 polarization, aiding OSCC immune evasion (98). Beyond direct effects on macrophages, these dysbiotic microbes and M2-polarized TAMs also profoundly shape the wider immune landscape of the OSCC tumor microenvironment. TAM-derived IL-10, TGF-β, and chemokines drive the recruitment and expansion of regulatory T cells (Tregs), which further suppress effector T cell function. Similarly, TAM-secreted factors such as CCL2 and CSF-1 promote the accumulation of myeloid-derived suppressor cells (MDSCs), reinforcing an immunosuppressive milieu. In parallel, persistent inflammatory signaling and PD-L1 expression by TAMs induce functional exhaustion of CD8^+^ cytotoxic T lymphocytes, characterized by upregulation of inhibitory receptors (PD-1, TIM-3, TIGIT) and diminished cytotoxicity. These interconnected suppressive networks, orchestrated by microbial signals and macrophage-derived mediators, contribute to a deeply immune-evasive tumor niche that fosters OSCC progression and resistance to therapy. Microbial-infected macrophages play a pivotal role in tumor development, providing insights for early oral cancer diagnosis and treatment (**Figure 1**). Recent clinical investigations have shown that the presence of dysbiotic microbial communities, particularly *Fusobacterium nucleatum* and *Porphyromonas gingivalis*, correlates with higher tumor stage, lymph node metastasis, and poorer survival outcomes in OSCC patients (111, 112). Elevated infiltration of M2-polarized TAMs in biopsy specimens is consistently associated with immune evasion, advanced stage disease, and resistance to immune checkpoint inhibitors (113, 114). These findings underscore the potential for using the oral microbiota composition and TAM-related markers as prognostic biomarkers, which also provide a rationale for integrating microbiota modulation and TAM-targeting therapies into clinical strategies to improve immunotherapy efficacy and survival in OSCC patients.

## 5 Conclusion

The intricate interplay between dysbiotic oral microbiota and TAMs—innate immune sentinels—shapes a profoundly immunosuppressive tumor microenvironment in OSCC. Pathogenic bacteria such as *Fusobacterium nucleatum* and *Porphyromonas gingivalis* promote chronic inflammation, epithelial-mesenchymal transition, and M2 polarization of macrophages via pattern recognition receptors and cytokine signaling. These M2-like TAMs, in turn, sustain immune evasion, angiogenesis, and tumor progression.

Currently, clinical interventions that directly target the oral microbiota–immune axis in OSCC remain limited. Approaches such as probiotics or oral microbiome modulation are under early investigation for improving periodontal health and reducing chronic inflammation, but robust evidence in OSCC prevention or treatment is lacking. Similarly, antibiotics have been explored for altering microbial communities, yet their nonspecific effects and potential to disrupt beneficial bacteria limit their clinical utility. Fecal microbiota transplantation has shown promise in gastrointestinal cancers for modulating systemic immunity, but its application in head and neck cancers, including OSCC, remains largely unexplored and without clinical validation.

These gaps underscore the need for carefully designed trials to evaluate whether microbiome-focused interventions can complement immunotherapy or standard treatments in OSCC. Future therapeutic strategies should prioritize targeting this microbiome–innate immunity axis. Interventions that reprogram TAMs toward a pro-inflammatory M1 phenotype or modulate microbial composition may restore antitumor immunity. In-depth mechanistic studies and clinical validation of dual-targeted therapies, focusing on innate immune modulation and microbiota regulation, could pave the way for precision immunotherapy in OSCC.
